# The *gad*B pathway confers oxidative stress tolerance in *Lactiplantibacillus plantarum* SAL and acts synergistically with L-glutamate to restore intestinal barrier integrity

**DOI:** 10.3389/fnut.2026.1909350

**Published:** 2026-09-21

**Authors:** Jie Sun, Wenqi Zhao, Jinqing Fan, Xinyi Lin, Jinlian Huang, Yamin Chen, Yanping Xu, Yuzhen Xu, Jiaxiao Sun, Xueying Cheng, Ben Liu, Mintao Zhong, Yongzhi Lun

**Affiliations:** 1 Key Laboratory of Screening and Control of Infectious Diseases, Fujian Provincial University, Quanzhou Medical College, Quanzhou, China; 2 Department of Clinical Laboratory, Tianjin Heping District Center for Disease Control and Prevention, Tianjin, China; 3 Department of Clinical Laboratory, The Affiliated Hospital of Putian University, Putian, China; 4 Department of Microbiology, School of Basic Medical Sciences, Guilin Medical University, Guilin, China; 5 Department of Anesthesiology, The First Hospital of Quanzhou Affiliated to Fujian Medical University, Quanzhou, China; 6 Department of Pathogen Biology and Microecology, School of Basic Medical Sciences, Dalian Medical University, Dalian, China

**Keywords:** glutamate decarboxylase, intestinal barrier, *Lactiplantibacillus plantarum*, oxidative stress, synbiotic

## Abstract

**Introduction:**

The therapeutic efficacy of probiotics is often limited by poor survival under gastrointestinal oxidative stress; however, stress adaptation mechanisms in *Lactiplantibacillus* remain poorly understood. This study evaluated the probiotic properties of a host-derived strain, *Lactiplantibacillus plantarum* SAL, elucidated the molecular basis of its oxidative stress tolerance, and assessed whether L-glutamate (L-Glu) supplementation synergistically enhances its protective effects on intestinal barrier integrity.

**Methods:**

Probiotic traits (gastrointestinal tolerance, antibacterial activity) and antitumor efficacy (CT26 colon cancer xenograft model in nude mice) of *L. plantarum* SAL were assessed. Transcriptome profiling of H_2_O_2_-challenged SAL identified key stress-responsive genes. The effects of L-Glu supplementation on oxidative stress resistance and antibacterial activity were evaluated *in vitro*. A D-galactose (D-gal)-induced oxidative injury mouse model was used to investigate the restorative effects of the *L. plantarum* SAL + L-Glu synbiotic on intestinal barrier function.

**Results:**

Compared with the reference strain *L. plantarum* WCFS1, SAL possessed superior probiotic traits, with significantly higher viability at 6% NaCl (*p* < 0.01) and in simulated gastric fluid at pH 1.5 after 2 h (*p* < 0.001). SAL administration markedly suppressed tumor growth and attenuated splenomegaly in the colon cancer model, with inhibition rates of 21.26% by tumor weight and 32.51% by tumor volume. Transcriptomic profiling identified 313 differentially expressed genes (149 upregulated, 164 downregulated), revealing glutamate decarboxylase (*gad*B) as a key oxidative stress tolerance gene. Heterologous expression of recombinant *Gad*B confirmed its enzymatic activity (14.06 U/mg), mediating resistance via γ-aminobutyric acid (GABA) biosynthesis. Supplementation with 1.5% L-Glu significantly increased the antioxidative capacity of *L. plantarum* SAL, maintaining viable counts at ~10^8^ CFU/mL under 8 mM H_2_O_2_ versus ~10^7^ CFU/mL without supplementation. In the D-gal-induced oxidative damage model, the *L. plantarum* SAL + L-Glu synbiotic restored colon barrier integrity, increased goblet cell numbers, reduced inflammatory infiltration, and decreased plasma levels of malondialdehyde (MDA) and oxidized glutathione (GSSG) to levels comparable to those of normal controls (*p* > 0.05). Mechanistically, *GadB*-mediated GABA biosynthesis conferred cross-protection against oxidative stress, a function distinct from its canonical acid resistance role and previously unrecognized in *Lactiplantibacillus*.

**Conclusion:**

Augmentation of the *gad*B pathway by exogenous L-Glu enhances the probiotic efficacy of *L. plantarum* SAL by synergistically boosting oxidative stress tolerance and reinforcing intestinal barrier repair. These findings establish *gad*B-mediated GABA biosynthesis as a previously unrecognized cross-protective mechanism against oxidative stress, thereby extending the classical acid-resistance paradigm of the GAD system. This targeted metabolic modulation thus offers a promising synbiotic strategy for counteracting oxidative stress-linked gut disorders. Although Nrf2 activation is a plausible mediator, direct confirmation in intestinal tissues and dose optimization are still required.

## 1 Introduction

Oxidative stress, driven by an imbalance between excessive reactive oxygen species (ROS) production and impaired clearance mechanisms (1), is a hallmark physiological response to environmental toxins, inflammation, and metabolic disturbances (2). ROS cause damage by oxidizing DNA, lipids, and proteins, thereby disrupting cellular structures and activating inflammatory cascades that ultimately lead to tissue dysfunction and apoptosis. Thus, interventions that mitigate oxidative stress and preserve gut redox homeostasis are of significant therapeutic interest.

Probiotics have attracted considerable interest for their roles in free radical scavenging, immune modulation, and intestinal barrier maintenance (3). *Lactiplantibacillus plantarum*, a well-recognized probiotic species, has demonstrated multiple health-promoting functions, including modulation of the gut microbiota, inhibition of pathogenic bacterial growth, and prevention of intestinal inflammation (4). However, to exert these beneficial effects, probiotics must survive gastrointestinal transit by resisting gastric acid, bile salts, and pancreatic enzymes, and subsequently colonize the gut to compete effectively with the resident microbiota (5, 6). Thus, the therapeutic efficacy of probiotics is often constrained by poor survival under the oxidative stress conditions prevalent in the gut.

Despite extensive research on *L. plantarum*, the molecular mechanisms underlying oxidative stress adaptation remain poorly understood (7). In particular, three knowledge gaps persist: (i) the genes and pathways conferring oxidative stress tolerance in host-derived *L. plantarum* strains remain uncharacterized; (ii) although the GAD system is canonically recognized as an acid resistance mechanism, its role in oxidative stress tolerance, particularly via the GABA shunt, remains largely unexplored in probiotic lactobacilli (8, 9); and (iii) synbiotic strategies based on targeted metabolic substrates that augment stress-response pathways remain nascent (10).

*L. plantarum* SAL, isolated from a patient with severe intestinal polyposis and high oxidative stress, exhibits inherent resilience, a premise supported by prior genomic and safety data. This study tests the hypothesis that the *gad*B-GABA pathway confers cross-protection against oxidative stress beyond its classical acid-resistance function, and that exogenous L-glutamate (L-Glu), the substrate for *Gad*B, can synergistically enhance this protective effect. The novelty of this work has three key aspects: (i) systematic elucidation of the *gad*B-mediated oxidative stress tolerance mechanism in a host-derived strain; (ii) demonstration that *Gad*B confers cross-protection against oxidative stress rather than acid alone; and (iii) development of a substrate-targeted synbiotic strategy (SAL + L-Glu) that differs from conventional probiotic-prebiotic combinations.

This study was designed as a three-tiered investigation, progressing from phenotypic characterization to mechanistic dissection and culminating in translational validation. Module 1 evaluated the probiotic properties and antitumor activity of *L. plantarum* SAL in a colon cancer xenograft model (phenotypic characterization). Module 2 employed transcriptomic profiling and heterologous expression in *E. coli* to identify and validate the function of *gad*B (mechanistic dissection). Module 3 tested whether L-Glu supplementation synergistically enhances the antioxidative capacity of SAL via the *gad*B pathway, and verified the protective effects of the SAL + L-Glu synbiotic on intestinal barrier integrity in a D-galactose-induced oxidative damage model (translational validation).

## 2 Materials and methods

### 2.1 Bacterial strains and cell culture

*Lactiplantibacillus plantarum* SAL, isolated and patented by the research group, is deposited at the China General Microbiological Culture Collection Center (CGMCC) under accession number 21786. The reference strain *L. plantarum* WCFS1 was purchased from Hangzhou Biosaier Biotechnology Co., Ltd. Indicator strains (*Escherichia coli* ATCC 25922, *Staphylococcus aureus* ATCC 25923, *Klebsiella pneumoniae* ATCC 700603, *Enterococcus faecalis* ATCC 29212, *Salmonella Typhimurium*, and *Proteus vulgaris*) were maintained by the research group.

The CT26 mouse colon cancer cell line (Chinese Academy of Sciences Shanghai Cell Bank) was cultured in RPMI-1640 medium (Thermo Fisher Scientific, USA) supplemented with 10% fetal bovine serum and 1% penicillin–streptomycin at 37 °C in a humidified atmosphere containing 5% CO_2_.

### 2.2 High-salt tolerance assay

MRS broth (6 mL) was prepared with NaCl concentrations ranging from 0 to 8% (w/v), including 1, 2, 4, and 6%. Suspensions of *L. plantarum* SAL and *L. plantarum* WCFS1 were inoculated (10% v/v) into NaCl-supplemented broths and incubated at 37 °C with shaking (150 rpm) for 20 h. Viable counts (log_10_ CFU/mL) were determined using the drop-plate method: 200 μL of each culture was serially diluted tenfold in sterile physiological saline. Subsequently, 20 μL aliquots of appropriate dilutions were spotted onto MRS agar plates. After incubation at 37 °C for 20 h, the colonies were counted. Three independent biological replicates (cultures initiated from separate colonies) were used. For each biological replicate, viable counts (log_10_ CFU/mL) were determined in technical duplicate using the drop-plate method, and the mean value was calculated. The dataset for each condition thus comprises *n* = 3 biologically independent values.

### 2.3 Bile salt tolerance assay

Bacterial suspensions were inoculated (10% v/v) into MRS broth containing 0.1–0.5% bile salts and incubated at 37 °C with shaking (150 rpm) for 20 h. Viable counts (log_10_ CFU/mL) were determined using the drop-plate method.

### 2.4 Simulated gastric fluid tolerance assay

Simulated gastric fluid was prepared by dissolving pepsin (Sangon Biotech, China) (1 g/100 mL) in acidic solutions adjusted to pH 1.5, 2.0, and 2.5. The solutions were filter-sterilized (0.22 μm). Bacterial suspensions were inoculated (10% v/v) into simulated gastric fluid and incubated at 37 °C with shaking (150 rpm). The cultures were sampled at 1 h, 2 h, and 4 h post-inoculation. Viable counts (log_10_ CFU/mL) were determined using the drop-plate method.

### 2.5 Simulated intestinal fluid tolerance assay

Simulated intestinal fluid was prepared by dissolving 3.4 g of KH_2_PO_4_ in distilled water, adjusting the pH to 6.8, and making up the volume to 250 mL. Trypsin (Solarbio, China) was added to a final concentration of 1% (w/v), and the solution was filter-sterilized (0.22 μm) and aliquoted. Suspensions of *L. plantarum* SAL and *L. plantarum* WCFS1 were inoculated (10% v/v) and incubated at 37 °C with shaking (150 rpm). The cultures were sampled at 2 h, 4 h, and 6 h post-inoculation. Viable counts (log_10_ CFU/mL) were determined using the drop-plate method.

### 2.6 Antibacterial activity assay

Cultures of *L. plantarum* SAL and *L. plantarum* WCFS1 at the late-logarithmic growth phase were spotted (2 μL per spot) onto MRS agar plates and incubated at 37 °C for 12 h to form lawns. Indicator strains were adjusted to a turbidity equivalent to 3.0 McFarland standard (approximately 9 × 10^8^ CFU/mL) in physiological saline. For each indicator strain, 200 μL of the suspension was mixed with 15 mL of molten soft agar (0.7% [w/v] agarose in the respective broth) and poured onto the *L. plantarum*-spotted MRS plates. The plates were incubated aerobically at 37 °C for 12 h. The inhibition zones surrounding the *L. plantarum* lawns were measured.

### 2.7 Establishment of a colon cancer xenograft model in nude mice

*L. plantarum* SAL and *L. plantarum* WCFS1 were activated twice on MRS agar plates (37 °C, 5% CO_2_, 20 h). Single colonies were inoculated into MRS broth and incubated anaerobically at 37 °C for 20 h. The bacterial cells were subsequently washed with sterile physiological saline and resuspended at a concentration of 1.0 × 10^9^ CFU/mL.

Animal experiments were conducted in accordance with the UK Animals (Scientific Procedures) Act 1986 and associated guidelines. The experimental protocol was approved by the Ethics Committee of Putian University (no. 2022-11). Forty specific pathogen-free (SPF) male BALB/c nude mice (body weight 22 ± 2–3 g) were purchased from Chongqing Ensiwei Biotechnology Co., Ltd. Mice were housed at 23–25 °C under a 12 h light/dark cycle with free access to food and water. After one week of acclimatization, mice were randomly assigned to four groups (*n* = 10 per group) using a computer-generated random number sequence (Excel RAND function). Group assignments and treatments were as follows: (i) blank control group, receiving daily oral gavage of sterile physiological saline (0.2 mL/mouse); (ii) tumor-bearing control group, receiving daily oral gavage of sterile physiological saline (0.2 mL/mouse); (iii) SAL group, receiving daily oral gavage of *L. plantarum* SAL suspension (1.0 × 10^9^ CFU/mL, 0.2 mL/mouse); and (iv) WCFS1 group, receiving daily oral gavage of *L. plantarum* WCFS1 suspension (1.0 × 10^9^ CFU/mL, 0.2 mL/mouse). Gavage was administered daily for 4 weeks. During the third week, CT26 cells in the logarithmic growth phase were harvested and prepared as a suspension (1 × 10^6^ cells/mL). Mice in the tumor-bearing control, SAL, and WCFS1 groups were subcutaneously injected with 200 μL of the cell suspension into the right axilla to establish colon cancer xenografts.

### 2.8 Assessment of the antitumor effects of *Lactiplantibacillus plantarum* SAL *in vivo*

Following tumor inoculation, the general health status (fur condition, activity, responsiveness) of the nude mice was monitored daily. Body weight, water intake, and food consumption were recorded every two days. The tumor length (L) and width (W) were measured weekly, and the tumor volume was calculated as (L × W^2^)/2. Two days after the final treatment, the mice were anesthetized by intraperitoneal injection of sodium pentobarbital (2.5 mg/kg, 0.2 mL/mouse). The tumors and spleens were excised and weighed, and the tumor volumes were measured. The tumor inhibition rate was calculated as follows: [(average tumor weight of the control group−average tumor weight of the treatment group)/average tumor weight of the control group] × 100%. The spleen index was calculated as the spleen weight (mg)/body weight (g). For each group, ten mice (*n* = 10) were used to evaluate tumor volume, tumor weight, and the spleen index. Among these, three mice were randomly selected for serum cytokine analysis (ELISA) and protein extraction from tumor tissues (Western blot), representing biological replicates (*n* = 3) for these molecular analyses.

### 2.9 Western blot

Total protein was extracted using RIPA lysis buffer (Beyotime, China) according to the manufacturer’s instructions. The protein concentration was determined using a BCA assay kit (Beyotime, China). Equal amounts of protein (20 μg) were separated by 10% SDS-PAGE and transferred onto PVDF membranes. The membranes were blocked with 5% skim milk at room temperature for 2 h, followed by incubation with primary antibodies against mouse Bax, Bcl-2, Caspase-3, and GAPDH (1:1000 dilution; Abcam, United States), and subsequently with an HRP-conjugated goat anti-rabbit IgG secondary antibody (1:1000 dilution; Beyotime, China). The protein bands were visualized using a chemiluminescence detection kit (Thermo Fisher Scientific, USA) and imaged. The band intensities were quantified using ImageJ software. Relative protein expression was calculated as the intensity of the target protein band normalized to the intensity of the corresponding GAPDH band.

### 2.10 ELISA assay

Blood was collected via retro-orbital puncture and allowed to clot for 2 h at room temperature. Serum was separated by centrifugation and stored at −80 °C until analysis. Serum levels of interferon-γ (IFN-γ), tumor necrosis factor-α (TNF-α), and tumor necrosis factor-β (TNF-β) were quantified using commercial ELISA kits (CUSABIO, China) following the manufacturer’s protocols.

### 2.11 Establishment of the *Lactiplantibacillus plantarum* SAL oxidative stress model

A suspension of *L. plantarum* SAL in sterile physiological saline was inoculated (3% v/v) into 200 mL MRS broth and incubated at 37 °C for 4 h. The culture was aliquoted (15 mL per tube). Different volumes of 30% H_2_O_2_ solution were added to achieve final concentrations of 0, 1, 2, 3, 4, 5, 6, 7, 8, 9, and 10 mM H_2_O_2_. Tubes were incubated statically at 37 °C for 0.5 h. Immediately after H_2_O_2_ treatment, bacteria were harvested by centrifugation (4,000 rpm, 10 min), washed twice with sterile physiological saline to remove residual H_2_O_2_, and resuspended in an equal volume of saline. Viable counts (log_10_ CFU/mL) were then determined using the drop-plate method to avoid interference from residual H_2_O_2_. The H_2_O_2_ concentration that induced growth stagnation was selected as the final stress condition.

### 2.12 Transcriptome sequencing

Total RNA was extracted from bacterial pellets using RNA TRIzol Reagent (Invitrogen, United States) according to the manufacturer’s instructions. Ribosomal RNA (rRNA) was depleted using the RiboMinus Transcriptome Isolation Kit (Bacteria) (Invitrogen, United States). Enriched mRNA was purified using a concentration module kit (Invitrogen, United States). The RNA concentration was measured using a Qubit fluorometer (Invitrogen, United States), and the purity was assessed using a NanoDrop spectrophotometer (Thermo Fisher Scientific, United States). Library construction and sequencing (Illumina HiSeq 2,500/MiSeq) were performed by Chongqing Western Biotech Co., Ltd. Raw read quality was assessed using FastQC. Quality control, reference sequence alignment, and transcript assembly were performed on the qualified reads.

### 2.13 Screening of key genes and construction of the regulatory network

Differentially expressed genes (DEGs) under oxidative stress were identified using the following thresholds: |log_2_ (fold change)| ≥ 1.1, *p* value < 0.05, and adjusted *p* value (adj. p) < 0.05. DEGs were submitted to the Metascape database^1^ for Gene Ontology enrichment analysis (biological process, BP; molecular function, MF; and cellular component, CC) and KEGG pathway enrichment analysis. The enrichment parameters included a minimum overlap of ≥ 3 and a *p*-value cutoff ≤ 0.05, with the other options set to default values. DEGs were also submitted to the STRING online tool^2^ to construct a protein–protein interaction (PPI) network with the following parameters: network edge meaning: confidence; active interaction sources: experiments, databases; minimum required interaction score ≥ 0.7; hidden disconnected nodes in the network; and other options set to default.

The protein–protein interaction network was visualized using Cytoscape software (version 3.9.1). Densely connected network clusters were identified using the MCODE plugin with the following parameters: degree cutoff = 2, node score cutoff = 0.2, k-core = 3, and max depth = 100. Network topological properties were calculated using the CentiScape plugin. Hub genes were defined as nodes with a degree ≥ (mean + standard deviation), whereas bottleneck genes were defined as those with a betweenness centrality ≥ (mean + standard deviation). A Venn diagram online tool was used to identify genes common to the Core (from MCODE), Hub, and Bottleneck classifications as key genes. Key pathways associated with these genes are summarized. Finally, a visual regulatory network was constructed in Cytoscape to depict the interactions between key genes and their associated pathways.

### 2.14 Quantitative real-time PCR (qRT-PCR)

Primers for target genes (*pur*A and *gad*B) and the reference gene (16S rRNA) were designed based on NCBI GenBank reference sequences. The sequences of the primers used were as follows: *pur*A F: 5’-ACGGTCAACGCGTCCTATTT-3′, R: 5’-ACCGGCAACTGGGTTAGATG-3′; *gad*B F: 5’-GTGGTCCCAATGGATGAGCA-3′, R: 5’-TGATGCCCATGATACCGACG-3′; 16S rRNA F: 5’-GCTCGTGTCGTGAGATGTT-3′, R: 5’-TGTAGCCCAGGTCATAAGG-3′.

Total bacterial RNA was extracted using RNA TRIzol Reagent. cDNA synthesis was performed using the PrimeScript RT reagent Kit (TaKaRa, Japan). Genomic DNA was removed by incubating the reaction mixture (total RNA (10 pg-5 μg)), 5 × gDNA Eraser Buffer (up to 20 μL), gDNA Eraser (1 μL), and RNase-free ddH_2_O (up to 10 μL) at 42 °C for 2 min. Reverse transcription was performed by adding PrimeScript RT Enzyme Mix I (1 μL), 5 × PrimeScript Buffer 2 (4 μL), RT Primer Mix (up to 20 μL), and RNase-free dH_2_O (up to 20 μL), followed by inactivation at 85 °C for 5 s and 37 °C for 15 min. Real-time PCR was performed using TB Green Premix Ex Taq II (Tli RNaseH Plus) (TaKaRa, Japan). The reaction mixture (25 μL) contained TB Green Premix Ex Taq II (12.5 μL), PCR forward primer (1 μL), PCR reverse primer (1 μL), cDNA template (2 μL), and RNase-free dH_2_O (up to 25 μL). Thermal cycling conditions included initial denaturation at 95 °C for 2 min; 40 cycles of 95 °C for 10 s and 60 °C for 30 s; and a melt curve stage of 95 °C for 15 s, 60 °C for 1 min, and 95 °C for 15 s. Relative gene expression was calculated using the 2^-ΔΔCt^ method.

### 2.15 Induction, expression, and purification of recombinant *gad*B protein

The *gad*B gene sequence from *L. plantarum* SAL was codon optimized for *E. coli* expression. BamHI and XhoI restriction sites were added to the 5′ and 3′ ends, respectively. The optimized sequence was synthesized by Shanghai Sangon Biotech Co., Ltd., and cloned into the pSmart-I vector. The recombinant plasmid (pSmart-I-*gad*B) was transformed into *E. coli* BL21(DE3) competent cells. Transformants were selected on LB agar plates supplemented with 50 μg/mL kanamycin (Kan) (Sangon Biotech, China). Positive clones were inoculated into LB broth supplemented with Kan and incubated overnight at 37 °C with shaking (200 rpm). Overnight cultures were diluted (1:100) into 300 mL of fresh LB broth with Kan and incubated at 37 °C with shaking until the OD_600_ reached 0.6. Protein expression was induced by the addition of 0.8 mM IPTG (MACKLIN, China) and incubation for 4 h at 37 °C. Expression was confirmed by SDS-PAGE. Uninduced pSmart-I-*gad*B culture and IPTG-induced pSmart-I empty vector culture served as controls.

Induced cells were harvested by centrifugation (8,000 × g, 5 min, 4 °C), washed with PBS, centrifuged again, and resuspended in 150 mL of ddH_2_O. Cells were lysed by sonication (350 W, 2 s pulse on/4 s pulse off) on ice. The lysate was subsequently centrifuged (10,000 × g, 10 min, 4 °C). The pellet (inclusion bodies) was dissolved in binding buffer (50 mM Tris–HCl, 8 M urea, and 500 mM NaCl). The supernatant (soluble fraction) was retained. Recombinant protein expression in both fractions was assessed by SDS-PAGE. Recombinant protein with a 6 × His tag was purified from the soluble fraction using Ni-NTA affinity chromatography under denaturing conditions. The purified protein was analyzed by SDS-PAGE, and the concentration was determined using a BCA assay.

### 2.16 Assays of the activity of the *gad*B recombinant protein

The protein encoded by *gad*B (glutamate decarboxylase, GAD) specifically catalyzes the decarboxylation of L-Glu or L-monosodium glutamate (L-MSG) to produce γ-aminobutyric acid (GABA) and CO_2_. On this basis, the activity of the purified GAD was determined by measuring the generation of GABA from L-Glu (Sangon Biotech, China) using pre-column phenyl isothiocyanate (PITC) (MACKLIN, China) derivatization followed by high-performance liquid chromatography (HPLC).

GABA standard solutions (Sangon Biotech, China) (1, 2, 3, 4, and 5 mg/mL) were prepared. For derivatization, 500 μL of the GABA standard solution was mixed with 500 μL of 0.1 M PITC in acetonitrile. After mixing, 500 μL of 1 M triethylamine in acetonitrile was added. The mixture was incubated at 30 °C for 1 h. Afterward, 3 mL of acetonitrile (Aladdin, China) (50% v/v) and 5 mL of n-hexane (Sinopharm, China) were added. The mixture was vortexed and allowed to phase separate for 5 min. The lower aqueous phase was collected, filtered (0.22 μm), and subjected to HPLC analysis to generate a GABA standard curve (peak area vs. concentration).

HPLC analysis was performed using a Promosil C18 column (250 × 4.6 mm, 5 μm) with UV detection at 254 nm. The injection volume was 10 μL, and the column temperature was maintained at 25 °C. The mobile phase consisted of methanol (A, Sinopharm, China) and a mixture of tetrahydrofuran (Sinopharm, China): methanol: 0.05 M sodium acetate (MACKLIN, China) (pH 6.2) = 5: 75: 420 (v/v/v) (B), delivered at a flow rate of 1.0 mL/min under isocratic elution (A: B = 20: 80) for a total run time of 15 min. To minimize degradation of the phenylisothiocyanate (PITC)-derivatized phenylthiocarbamyl derivatives, the autosampler tray was maintained at 4 °C, and all derivatized samples were protected from light and analyzed within 2 h of preparation. Between consecutive injections, the column was re-equilibrated with the starting mobile phase composition for 5 min to ensure consistent retention times and peak area reproducibility. Reaction substrate: 0.05 mM pyridoxal 5′-phosphate (PLP) and 50 mM L-Glu in 0.2 M sodium acetate–acetic acid buffer (pH 4.8). Reaction: Five hundred microliters of prewarmed substrate (48 °C) was mixed with 100 μL of purified GAD recombinant protein. The mixture was incubated at 50 °C for 10 min. The reaction was terminated by boiling for 5 min. The supernatant was collected after centrifugation and analyzed by high-performance liquid chromatography (HPLC) to quantify GABA production. The GAD activity (U/mg) was calculated on the basis of the GABA production rate. A reaction with ddH_2_O instead of the enzyme served as the control. One unit (U) of GAD activity is defined as the amount of enzyme that catalyzes the formation of 1 μmol of GABA per minute under the assay conditions. Specific activity is expressed as U per mg of protein.

### 2.17 Antioxidant activity of the recombinant *gad*B expression strain

The antioxidant effect of *gad*B expression was evaluated by comparing the survival rates of *E. coli* expressing pSmart-I-*gad*B with those of the pSmart-I empty vector control under oxidative stress. *E. coli* cultures were inoculated into 80 mL of LB broth with Kan and incubated for 2 h. Expression was induced by adding 0.4 mM IPTG, followed by incubation for 4 h. The cultures were adjusted to a turbidity equivalent to 3.0 McFarland standard. Aliquots (2 mL) were dispensed into four centrifuge tubes. Different concentrations of 30% H_2_O_2_ were added to achieve final concentrations of 0 mM, 5 mM, 10 mM, and 15 mM. Tubes were incubated at 37 °C for 0.5 h. Immediately after incubation, serial 10-fold dilutions were prepared and plated to determine viable counts (log_10_ CFU/mL) using the drop-plate method, thereby minimizing the confounding effect of residual H_2_O_2_ on subsequent colony enumeration.

### 2.18 Effects of L-Glu on the oxidative stress resistance of *Lactiplantibacillus plantarum* SAL

*L. plantarum* SAL cultures at the late logarithmic growth phase were inoculated into MRS broth supplemented with varying L-Glu concentrations (0, 0.5, 1.0, 1.5, 2.0, and 3.0%). After incubation at 37 °C for 12 h, viable counts (log_10_ CFU/mL) were determined to determine the optimal L-Glu concentration.

Cultures of *L. plantarum* SAL incubated for 4 h and 12 h were centrifuged, and the supernatants were discarded. For each time point, eight tubes containing 15 mL MRS broth served as the control group. Another eight tubes containing 15 mL MRS broth with 1.5% L-Glu served as the 1.5% L-Glu group. Different concentrations of 30% H_2_O_2_ were added to each tube to achieve final concentrations of 0 mM, 2 mM, 3 mM, 4 mM, 5 mM, 6 mM, 7 mM, and 8 mM H_2_O_2_. Tubes were incubated at 37 °C for 0.5 h. Viable counts (log_10_ CFU/mL) were determined to assess the protective effect of L-Glu against oxidative stress.

### 2.19 Effects of L-Glu on the antibacterial activity of *Lactiplantibacillus plantarum* SAL

The effect of L-Glu on antibacterial activity was tested by measuring the inhibition zones produced. The *L. plantarum* SAL suspension was spotted onto MRS agar plates containing 1.5% L-Glu (1.5% L-Glu group) and standard MRS agar plates (control group). The plates were incubated at 37 °C for 12 h to form lawns. Two clinically relevant pathogens, *Klebsiella pneumoniae* (a Gram-negative bacillus) and *Enterococcus faecalis* (a Gram-positive coccus), were selected as indicator strains.

### 2.20 Establishment of an oxidative stress-induced mouse model

On the basis of the acceptable daily intake (ADI) of glutamate for humans (≤6 g/day for a 60 kg person), the daily intake for mice (30 g) was set at 3 mg L-Glu/day. A synbiotic solution containing *L. plantarum* SAL (1.0 × 10^9^ CFU/mL) and 1% (w/v) L-Glu in sterile physiological saline was prepared and aliquoted. This concentration (1%) was selected for *in vivo* administration on the basis of preliminary dose-finding studies and practical limitations of oral gavage volume, whereas the higher concentration (1.5%) used *in vitro* was chosen to maximize substrate availability under controlled culture conditions without adverse effects on bacterial growth. Fifty-six SPF male Kunming mice (body weight 22 ± 2–3 g) were purchased from Fuzhou Nuodunsi Biotechnology Co., Ltd. After one week of acclimatization, the mice were randomly divided into a normal control group (*n* = 13) and an experimental group (*n* = 43). The mice in the experimental group received daily intraperitoneal injections of D-galactose (D-gal, 2000 mg/kg in 0.2 mL saline) for two weeks (loading phase). Following confirmation of successful model establishment, the dosage was reduced to 1,000 mg/kg administered every other day for the subsequent four weeks (maintenance phase). The normal control group received equivalent volumes of sterile saline. Injections continued for two weeks. After two weeks, three mice per group were randomly selected. Plasma malondialdehyde (MDA) levels and total superoxide dismutase (T-SOD) activity (Elabscience, China) were measured. A statistically significant difference (*p* < 0.05) confirmed successful model establishment.

### 2.21 Effects of *Lactiplantibacillus plantarum* SAL + L-Glu synbiotic on oxidative stress-damaged mice

Following successful model establishment, the experimental group of mice was randomly subdivided into four groups (*n* = 10 per group). All groups received intraperitoneal injections of D-gal (1,000 mg/kg in 0.2 mL saline) every other day. The normal control group received equivalent sterile saline injections every other day. Different treatments were administered daily by oral gavage (0.1 mL/10 g body weight): the normal control group and D-gal model group received sterile saline; the SAL group received an *L. plantarum* SAL suspension (1.0 × 10^9^ CFU/mL); the L-Glu group received 1% L-Glu solution; and the Synbiotic group received *L. plantarum* SAL + 1% L-Glu synbiotic solution. Treatment was continued for 4 weeks. 12 h after the final gavage (fasted, water ad libitum), body weight was recorded. The mice were anesthetized by intraperitoneal injection of sodium pentobarbital (2.5 mg/kg, 0.2 mL/mouse). Blood was collected via retro-orbital puncture into heparinized tubes. The plasma was separated by centrifugation (3,000 × g, 15 min, 4 °C). Plasma levels of MDA, oxidized glutathione (GSSG), and T-SOD activity were measured using commercial kits (Elabscience, China) according to the manufacturer’s instructions. The mice were euthanized by cervical dislocation. The colon was excised, and its length was measured. Colon tissue from three randomly selected mice per group was fixed in 4% paraformaldehyde (Servicebio, China), embedded in paraffin, sectioned, dewaxed, rehydrated, stained with hematoxylin and eosin (H&E), dehydrated, and mounted for histopathological examination under light microscopy.

### 2.22 Statistical analysis

Data were analyzed using GraphPad Prism 9.5 and are presented as mean ± SD. *In vitro* data represent three biological replicates, each with three technical replicates. *In vivo* physiological parameters (tumor volume, spleen index, plasma markers) used *n* = 10 per group, whereas molecular analyses used *n* = 3 per group.

Normality and homogeneity of variance were assessed by Shapiro–Wilk and Brown-Forsythe tests, respectively. For two-group comparisons, unpaired two-tailed Student’s *t*-test was applied, with Welch’s correction when variances were unequal. For multi-group comparisons, parametric data were analyzed by one-way ANOVA followed by Tukey’s *post hoc* test (all pairwise) or Dunnett’s *post hoc* test (vs. control); when assumptions were violated, Kruskal-Wallis with Dunn’s test was used. Two-way ANOVA with Sidak’s *post hoc* test was used for two-factor designs. For repeated measures, sphericity was checked by Mauchly’s test, with Greenhouse–Geisser correction applied if violated. For multiple *t*-tests, *p*-values were adjusted using the Benjamini-Hochberg method. All tests were two-tailed, with significance defined as: ns (not significant, *p* > 0.05), * *p* < 0.05, ** *p* < 0.01, *** *p* < 0.001, **** *p* < 0.0001.

## 3 Results

### 3.1 Probiotic properties of *Lactiplantibacillus plantarum* SAL

After 20 h exposure to NaCl at concentrations up to 2 g/100 mL, viable counts of both *L. plantarum* SAL and WCFS1 decreased. However, further increases in NaCl concentration significantly increased viable counts for both strains, peaking at 6 g/100 mL NaCl. Notably, compared with WCFS1, *L. plantarum* SAL maintained markedly higher viable counts at 6% NaCl (*p* < 0.01) (Figure 1A).

**Figure 1:**
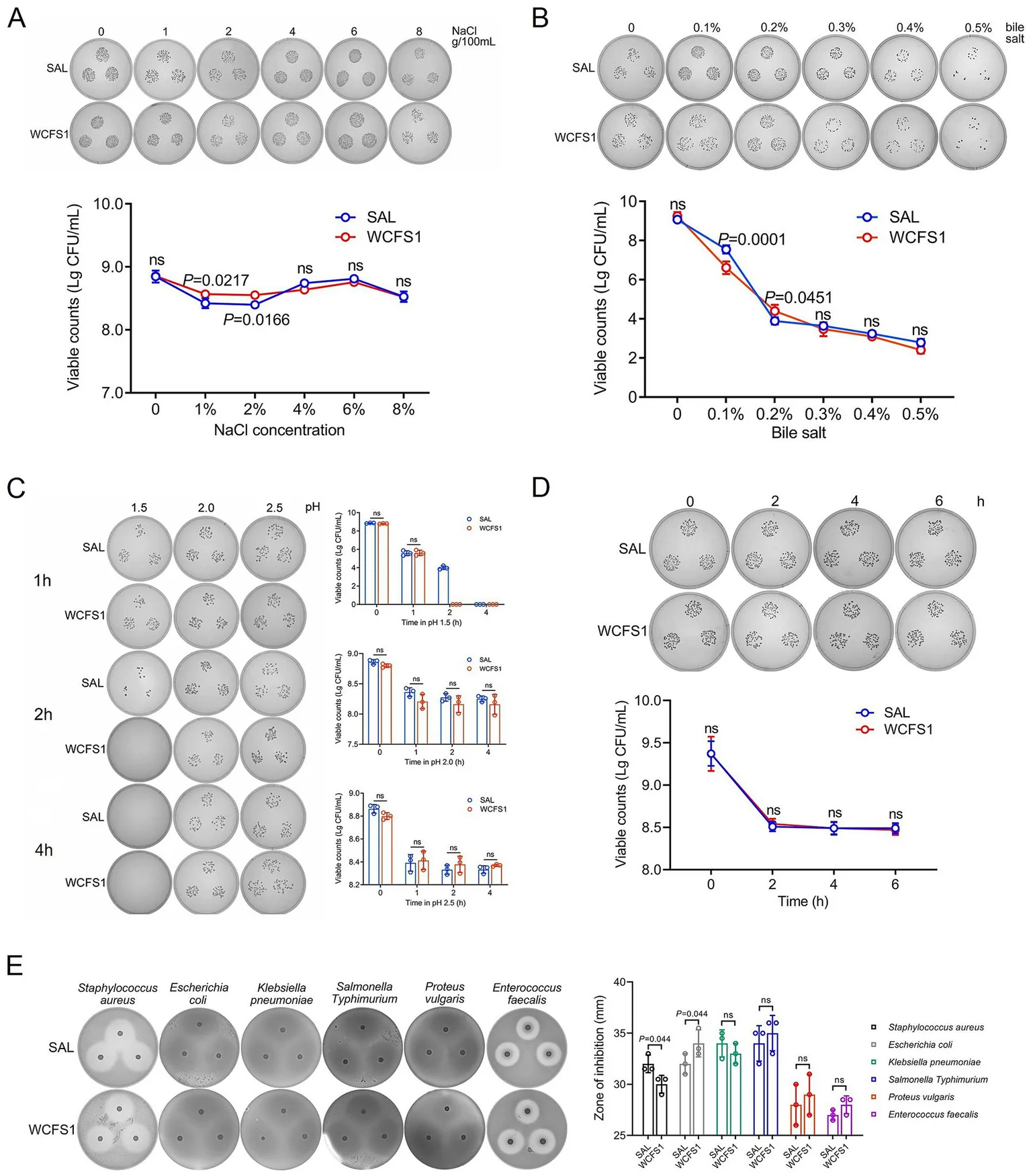
Probiotic properties of *Lactiplantibacillus plantarum* SAL compared with the reference strain *L. plantarum* WCFS1. **(A)** High salt tolerance at NaCl concentrations ranging from 0 to 8% (w/v). **(B)** Bile salt tolerance at concentrations ranging from 0 to 0.5% (w/v). **(C)** Tolerance to simulated gastric fluid at pH 1.5, 2.0, and 2.5. **(D)** Tolerance to simulated intestinal fluid (pH 6.8 with 1% trypsin). **(E)** Antibacterial activity against six indicator pathogens, measured as diameter of inhibition zones. Data are mean ± SD (*n* = 3). For **(A–D)**: Two-way ANOVA with Sidak’s test; for **(E)**: Unpaired *t*-tests with Benjamini-Hochberg correction. Significance: Ns, not significant (*p* > 0.05); *, *p* < 0.05; **, *p* < 0.01; ***, *p* < 0.001; ****, *p* < 0.0001.

The viable counts of both *L. plantarum* SAL and WCFS1 gradually decreased with increasing bile salt concentration. With 0.2% bile salts, the counts for SAL and WCFS1 decreased from 10^9^ CFU/mL to 10^3^–10^4^ CFU/mL and 10^4^ CFU/mL, respectively; the decrease was slightly more pronounced for SAL. At higher bile salt concentrations (>0.2%), the decrease in viable counts for both strains slowed, declining only from ~10^3^ to ~10^2^ CFU/mL, with comparable tolerance levels observed (Figure 1B).

After 1 h in simulated gastric fluid (pH 1.5), the viable counts of both *L. plantarum* SAL and WCFS1 decreased to 10^5^ CFU/mL. After 2 h, the SAL concentration was detectable at 10^3^ CFU/mL, while WCFS1 growth was below the detection limit, indicating significantly greater acid tolerance for SAL at this time point (*p* < 0.001). After 3 h, the growth of both strains was suppressed. These findings indicate that compared with WCFS1, *L. plantarum* SAL is more tolerant to simulated gastric fluid. In simulated gastric fluid at pH 2.0 and 2.5, viable counts for both strains remained at approximately 10^8^ CFU/mL (Figure 1C). After 2 h in simulated intestinal fluid, the viable counts for both *L. plantarum* SAL and WCFS1 decreased from 10^9^ CFU/mL to 10^8^ CFU/mL, although compared with WCFS1, SAL resulted in slightly better survival. With prolonged incubation, the viable counts stabilized for both strains, remaining above 10^8^ CFU/mL, indicating high and comparable tolerance to simulated intestinal fluid (Figure 1D).

Both *L. plantarum* SAL and WCFS1 exhibited significant antibacterial activity against all six indicator strains, resulting in inhibition zones larger than 27 mm in diameter. Both strains showed the strongest inhibitory effect against *Salmonella Typhi* and a slightly weaker effect against *Enterococcus faecalis*. Both strains showed comparable antibacterial activity against the six indicator strains, with no statistically significant difference between them (*p* > 0.05 for all comparisons). Both strains showed greater efficacy against Gram-negative than against Gram-positive bacteria (Figure 1E).

### 3.2 Effect of *Lactiplantibacillus plantarum* SAL on CT26 colon cancer cell tumorigenesis in nude mice

The experimental workflow is depicted in Figure 2A. Following modeling, the body weights of the nude mice bearing colon cancer xenografts continuously decreased. Compared with the blank control group (Ctrl), average body weight in the tumor-bearing control group decreased by 1.98 g, whereas weights in the SAL and WCFS1 groups decreased by only 0.84 g and 0.74 g, respectively, indicating that both strains mitigated tumor-induced weight loss (Figure 2B). Two weeks after tumor inoculation, the mice were sacrificed, and the tumors were excised and weighed. The tumor weights in the SAL and WCFS1 groups were markedly lower than those in the tumor control group, with inhibition rates of 21.26 and 24.98%, respectively (Figure 2C). Subcutaneous tumor growth velocity was markedly lower in the SAL and WCFS1 groups than in the tumor control group, with inhibition rates reaching 32.51 and 31.74%, respectively (Figure 2D). Spleen indices in the tumor ctrl, SAL, and WCFS1 groups were markedly greater than those in the Ctrl group but significantly lower in the SAL and WCFS1 groups than in the tumor ctrl group (Figure 2E). Serum levels of TNF-α, TNF-β, and IFN-γ showed no significant differences between the blank control and tumor-bearing control groups. In contrast, both the SAL and WCFS1 groups exhibited significantly elevated levels of these cytokines compared with the tumor-bearing control group (Figure 2F), indicating enhanced Th1-type immune activation.

**Figure 2:**
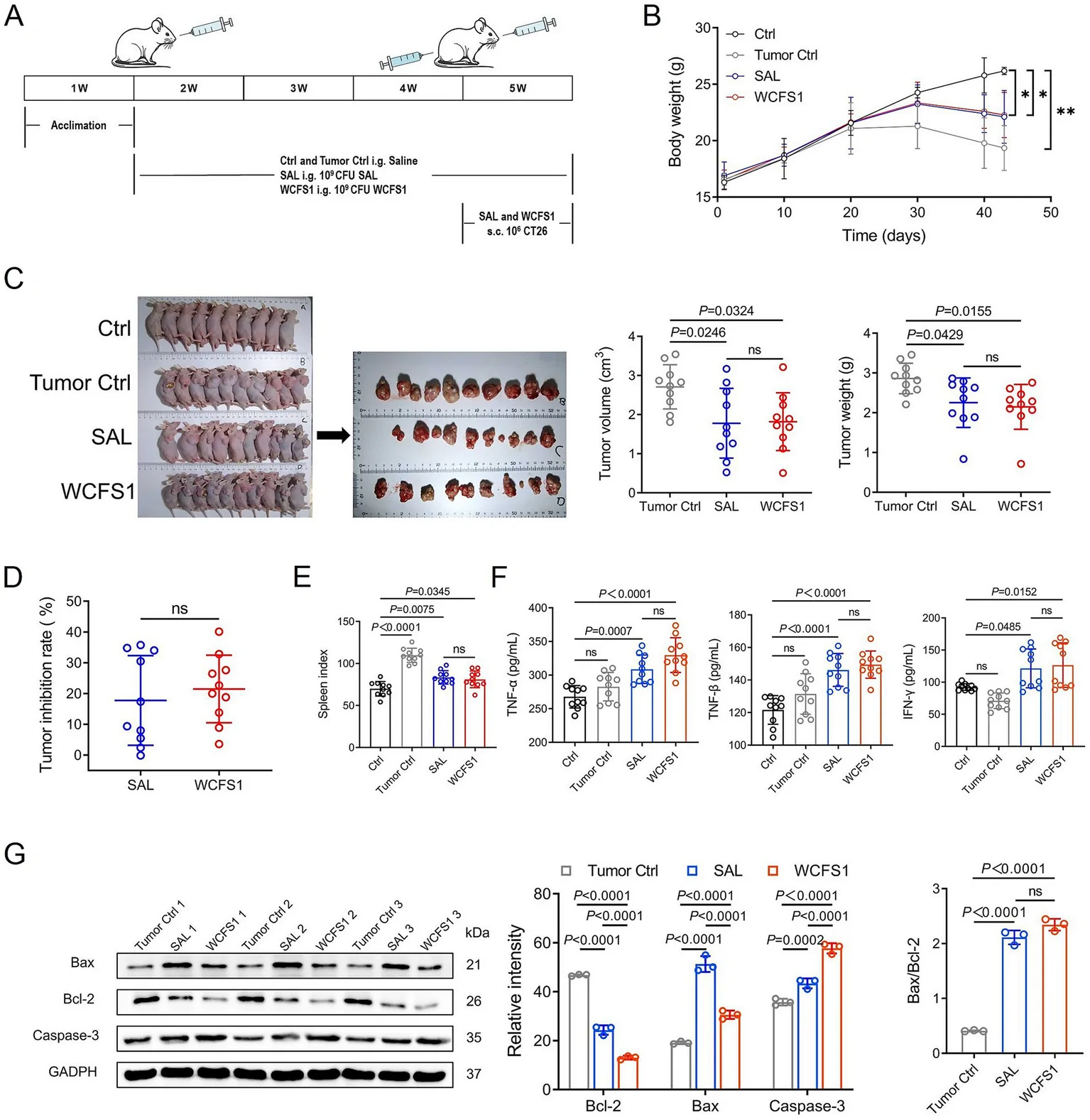
Antitumor effects of *L. plantarum* SAL in a CT26 colon cancer xenograft model in nude mice. **(A)** Experimental workflow. **(B)** Body weight changes over the 4-week treatment period. **(C)** Tumor weight and tumor volume at endpoint. **(D)** Tumor inhibition rate calculated as percentage reduction in tumor weight relative to the tumor control group. **(E)** Spleen index calculated as spleen weight (mg) per gram of body weight. **(F)** Serum levels of proinflammatory cytokines TNF-α, TNF-β, and IFN-γ measured by ELISA. **(G)** Western blot analysis of apoptosis-related proteins Bax, Bcl-2, and Caspase-3 in tumor tissues, with quantification of the Bax/Bcl-2 ratio. Data are mean ± SD (*n* = 10 for tumor/spleen parameters; *n* = 3 for cytokines and Western blot). For **(B)**: Two-way repeated-measures ANOVA with Sidak’s test (Greenhouse–Geisser correction if sphericity violated). For **(C–F)**: One-way ANOVA with Tukey’s test. For **(G)**: Unpaired *t*-test with Welch’s correction. Significance as in Figure 1.

The antitumor mechanism of *L. plantarum* SAL was explored by detecting endogenous apoptotic proteins. Compared with the tumor control group, the expression levels of Bax and Caspase-3 were significantly elevated, whereas Bcl-2 expression was significantly reduced in both the SAL and WCFS1 groups (Figure 2G), suggesting that *L. plantarum* SAL may exert its antitumor effect by promoting apoptosis (11). Although Caspase-3 expression appeared higher in the WCFS1 group than in the SAL group, this difference did not reach statistical significance (*p* > 0.05). The Bax/Bcl-2 ratio was comparable between the two treatment groups (*p* > 0.05). These results confirm that *L. plantarum* SAL possesses potent antitumor and immunomodulatory activities, which are desirable traits for a probiotic candidate. The following experiments were designed to investigate the molecular basis of its stress resistance, which is critical for its survival and functionality in the gut.

### 3.3 Regulatory mechanisms of *Lactiplantibacillus plantarum* SAL in response to oxidative stress

*L. plantarum* SAL cultured to the logarithmic phase was treated with H_2_O_2_. Low concentrations of oxidative stimulus did not inhibit growth. Viable bacterial counts began to decline when the H_2_O_2_ concentration reached 3 mM. However, bacterial viability remained stable (9.1–9.4 log_10_ CFU/mL) when the H_2_O_2_ concentration ranged from 0 to 5 mM. A more statistically significant difference compared with the untreated control emerged at concentrations above 6 mM. At H_2_O_2_ concentrations ≥ 8 mM, parallel samples showed substantial variability (Figure 3A). On the basis of these results, treatment with 5 mM H_2_O_2_ for 0.5 h was selected to establish an oxidative stress model for *L. plantarum* SAL. This concentration induced a mild but consistent reduction in viability, allowing for the capture of transcriptional responses associated with oxidative stress adaptation rather than acute lethality, which is appropriate for identifying key stress-responsive genes. Of note, oxidative stress caused by H_2_O_2_ is known to lower intracellular pH in many bacteria (e.g., through the Fenton reaction and membrane damage), which may secondarily activate acid-responsive pathways such as the *gad*B-encoded GAD system (12). Thus, the observed upregulation of *gad*B likely reflects a cross-protection mechanism.

**Figure 3:**
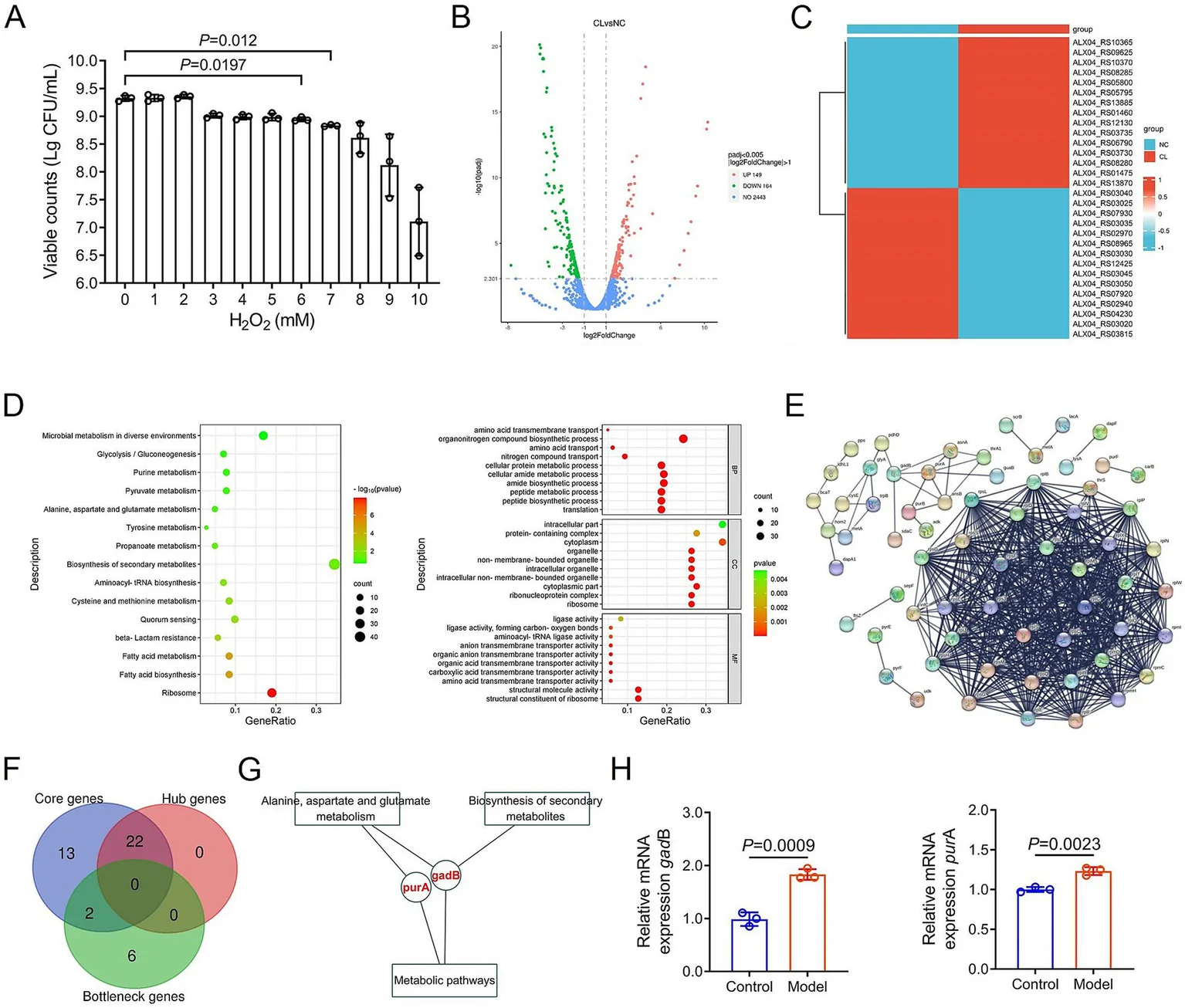
Transcriptomic analysis of *L. plantarum* SAL under H_2_O_2_-induced oxidative stress. **(A)** Viable counts of *L. plantarum* SAL after 0.5 h exposure to increasing concentrations of H_2_O_2_ (0–10 mM). **(B)** Volcano plot showing differentially expressed genes (DEGs) between the control group (NC) and the H_2_O_2_-treated group (CL). **(C)** Hierarchical cluster analysis of the top 30 upregulated and downregulated DEGs. **(D)** Gene Ontology (biological process, cellular component, and molecular function) and KEGG pathway enrichment analysis of DEGs. **(E)** Protein–protein interaction (PPI) network of DEGs constructed using the STRING database. **(F)** Venn diagram identifying key genes at the intersection of core, hub, and bottleneck gene classifications. **(G)** Regulatory network linking key genes (*gad*B and *purA*) to enriched KEGG pathways. **(H)** qPCR validation of *gad*B and *purA* expression levels relative to the 16S rRNA reference gene. Data are mean ± SD (*n* = 3). For **(A)**: One-way ANOVA with Dunnett’s test vs. 0 mM; for **(H)**: Unpaired *t*-test with Welch’s correction. Significance as in Figure 1.

The 5 mM H_2_O_2_ challenge for 0.5 h was selected on the basis of preliminary kinetic assays, which indicated that this exposure induced a modest but reproducible reduction in viability (approximately 0.3 log_10_ CFU/mL) without causing widespread cell death. This sublethal condition was chosen to capture early transcriptional reprogramming events associated with oxidative stress adaptation rather than acute lethal responses, thereby increasing the likelihood of identifying regulatory hubs such as *gad*B that prime the cell for enhanced tolerance.

Transcriptome profiling was performed under a sublethal oxidative challenge to delineate the underlying regulatory circuitry. The *L. plantarum* SAL wild-type group was designated the control group (NC), and the H_2_O_2_-treated group was designated the experimental group (CL). Transcriptome sequencing libraries were constructed and subjected to high-throughput sequencing. The NC and CL groups yielded 7,669,420 and 7,326,248 high-quality clean reads, respectively. The GC content was 44.19% (NC) and 44.84% (CL). The Pearson correlation coefficient (R^2^ > 0.8) indicated high consistency between replicate samples. Although the H_2_O_2_-treated group yielded fewer clean reads than the control group did, the Pearson correlation coefficient indicated high consistency between replicate samples. Read counts were normalized using the DESeq2 median-of-ratios method prior to differential expression analysis to minimize bias introduced by sequencing depth differences. A total of 313 differentially expressed genes (DEGs) were identified (Figure 3B), consisting of 149 upregulated genes and 164 downregulated genes. The top 30 significantly differentially expressed genes clearly clustered into two groups (Figure 3C). DEGs were enriched in several Gene Ontology terms, including biological processes (e.g., transcription, polypeptide metabolism and synthesis, cellular amide biosynthesis, and protein metabolic process), cellular components (e.g., ribosome and nucleoprotein complex), and molecular functions (e.g., structural molecule activity and ribosomal structural molecule activity). KEGG pathway enrichment analysis revealed the involvement of the ribosome, fatty acid biosynthesis, fatty acid metabolism, beta-lactam resistance, and bacterial chemotaxis pathways (13) (Figure 3D).

The candidate gene set was uploaded to the STRING online tool and screened according to preset parameters (Figure 3E). Using the “MCODE” plugin with defined settings, 37 core genes were identified, resulting in the formation of two interaction networks with K-Cores ≥ 3. The CentiScape plugin was used to calculate network topological features, identifying 22 hub genes (degree value ≥ mean + SD) and 8 bottleneck genes (betweenness value ≥ mean + SD). Genes simultaneously classified as core, hub, and bottleneck genes are crucial for the regulatory network. In the absence of such overlap, the intersection of at least two categories was taken. The intersection of core and hub genes yielded 22 genes, all encoding bacterial ribosomal proteins. The intersection of the core and bottleneck genes revealed two upregulated genes: glutamate decarboxylase (*gad*B) and adenylosuccinate synthase (*pur*A) (Figure 3F). As key genes that regulate oxidative stress in *L. plantarum* SAL, *gad*B and *pur*A participate in metabolic pathways, specifically alanine, aspartate and glutamate metabolism. *gad*B is also involved in the biosynthesis of secondary metabolites (14) (Figure 3G). qPCR validation confirmed that the relative expression levels of *gad*B and *pur*A in bacterial cells were consistent with the transcriptome sequencing results and were significantly upregulated in the oxidative stress group. *gad*B exhibited more pronounced differential expression and was therefore selected as the primary key gene for subsequent studies (Figure 3H).

### 3.4 Functional characterization of recombinant GAD protein

The full-length *gad*B gene of *L. plantarum* SAL is 1,410 bp in length and encodes GAD, which is 469 amino acids in length. The gene sequence was optimized on the basis of the *E. coli* codon usage frequency, and sequences encoding BamHI and XhoI restriction sites were added to the 5′ and 3′ ends, respectively, resulting in a synthesized 1,419 bp BamHI-*gad*B-XhoI fragment. The pSmart-I prokaryotic expression vector contains a 6 × His tag sequence at both the 5′ and 3′ ends of its multiple cloning site, along with an N-terminal SUMO fusion tag, to ensure correct expression and enhance the purification efficiency of the recombinant protein (Figure 4A). Bacterial pellets from induced cultures of pSmart-I-*gad*B-positive clones were boiled for SDS-PAGE analysis. The predicted size of the SUMO-GAD fusion protein is 66.91 kDa. When protein from the empty pSmart-I vector was used as a control, both the induced and uninduced pSmart-I-*gad*B samples presented distinct bands at ~70 kDa, which aligned with expectations and confirmed successful expression of the SUMO-GAD recombinant protein (Figure 4B).

**Figure 4:**
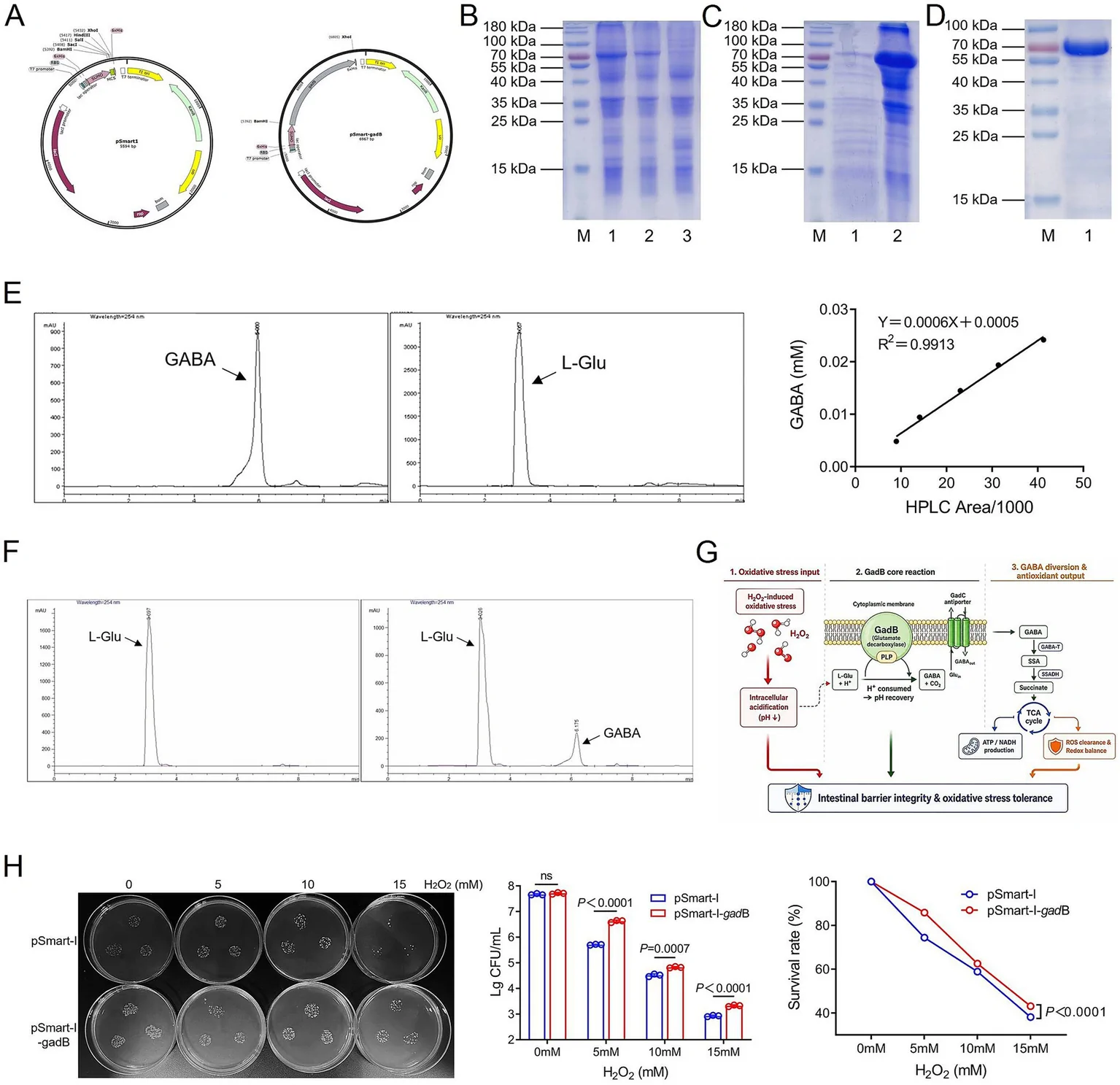
Functional characterization of the recombinant *Gad*B protein. **(A)** Schematic representation of the pSmart-I-*gad*B recombinant plasmid construct. **(B)** SDS-PAGE analysis of SUMO-GAD fusion protein expression. M: Pre-stained protein ladder; Lane 1: pSmart-I-*gad*B (IPTG-induced); Lane 2: pSmart-I-*gad*B (uninduced); Lane 3: pSmart-I empty vector (IPTG-induced). **(C)** SDS-PAGE analysis of SUMO-GAD expression in soluble (supernatant) and insoluble (inclusion body) fractions. **(D)** SDS-PAGE analysis of the purified SUMO-GAD protein. **(E)** HPLC chromatograms of GABA and L-Glu standards after pre-column derivatization with phenyl isothiocyanate. **(F)** HPLC detection of GABA production catalyzed by purified SUMO-GAD; ddH_2_O instead of enzyme served as the control. **(G)** Schematic illustration of the *Gad*B/GABA shunt pathway. Under H_2_O_2_-induced oxidative stress, intracellular acidification promotes *gad*B transcription. *Gad*B decarboxylates L-Glu to GABA, which enters the TCA cycle via the GABA shunt, supporting ATP production and redox balance. PLP: Pyridoxal 5′-phosphate; GABA: γ-aminobutyric acid; SSA: Succinic semialdehyde; SSADH: Succinate semialdehyde dehydrogenase. **(H)** Oxidative stress tolerance of *E. coli* strains expressing pSmart-I (empty vector control) or pSmart-I-*gad*B, assessed by viable counts after 0.5 h exposure to increasing concentrations of H_2_O_2_ (0–15 mM). Data are mean ± SD (*n* = 3). For H, two-way ANOVA with Sidak’s test. Significance as in Figure 1.

A gradient of IPTG induction concentrations (0.1–2 mM) was tested. Higher concentrations caused excessive cytotoxicity, whereas lower concentrations failed to saturate the LacI repressor and prolonged the induction time. Consequently, 0.8 mM IPTG induction for 4–6 h (sufficient for high expression by 3 h) was selected. Large-scale cultures were induced, and the cells were harvested and lysed by sonication (~15 min until the lysate was clarified). The soluble (supernatant) and insoluble (inclusion body) fractions were analyzed by SDS-PAGE, which revealed recombinant protein expression in both fractions (Figure 4C). Although inclusion bodies contain substantially higher protein levels, their denaturation makes renaturation difficult. Therefore, the soluble fraction was chosen for purification. SUMO-GAD, bearing a His-tag, was adsorbed onto Ni-NTA affinity resin and eluted. The purified protein produced a single band of the expected size (Figure 4D). A BCA assay revealed that the concentration of the purified recombinant protein was 0.202 mg/mL. GABA standard and L-Glu solutions were derivatized pre-columns and analyzed by HPLC to establish detection conditions using isocratic elution with 20% A and 80% B at a flow rate of 1.0 mL/min for a total run time of 15 min. Characteristic peaks for GABA and L-Glu appeared at retention times of ~5.90 min and 3.067 min, respectively, with the GABA peak area proportional to the concentration (Figure 4E). The GABA standard curve yielded the regression equation Y = 0.0006X + 0.0005 (R^2^ = 0.9913), confirming a reliable linear relationship between the GABA content and peak area.

Under optimal temperature and pH conditions and in the presence of the coenzyme pyridoxal 5′-phosphate (PLP), GAD efficiently catalyzes the decarboxylation of L-Glu. HPLC analysis of the reaction mixture containing recombinant SUMO-GAD, its substrate L-Glu, and PLP revealed two prominent chromatographic peaks corresponding to L-Glu and the product GABA. A control reaction with deionized water resulted in the absence of a GABA peak (Figure 4F). The GABA peak area (3896.06) was converted to a concentration using the standard curve, yielding 2.84 μM GABA produced by GAD catalysis. Enzyme activity (U/mg) was calculated as 14.06 U/mg on the basis of the standard definition (μmol product formed per minute per mg protein).

With 0 mM H_2_O_2_, the growth of the pSmart-I control strain and the pSmart-I-*gad*B recombinant strain was similar. With increasing H_2_O_2_ concentration, the resistance of the pSmart-I-*gad*B strain to oxidative stress significantly increased. In particular, under 5 mM H_2_O_2_, the survival rate of the recombinant strain far exceeded that of the control strain (Figure 4H). Although survival rates decreased as H_2_O_2_ concentration increased for both strains, viable counts of the pSmart-I-*gad*B strain consistently surpassed those of the control. These findings demonstrate that *gad*B expression confers oxidative stress tolerance in a heterologous host, extending the known function of the GAD system beyond acid resistance to include oxidant defense. This is a previously underappreciated aspect of this metabolic pathway.

### 3.5 Protective effect of L-Glu on *Lactiplantibacillus plantarum* SAL against oxidative stress

As the L-Glu concentration in the medium increased from 0 to 0.5%, the viable counts of *L. plantarum* SAL increased rather than decreased. The counts began to decrease when the L-Glu concentration reached 1.5%, although the order of magnitude (10^9^ CFU/mL) remained unchanged. Further increases in the L-Glu concentration resulted in a significant downward trend in growth (Figure 5A). To select a concentration without a significant negative impact compared with no L-Glu and adhering to the standard L-Glu usage level in food/industry (≤1.5%), 1.5% L-Glu was chosen as the optimal supplementation concentration.

**Figure 5:**
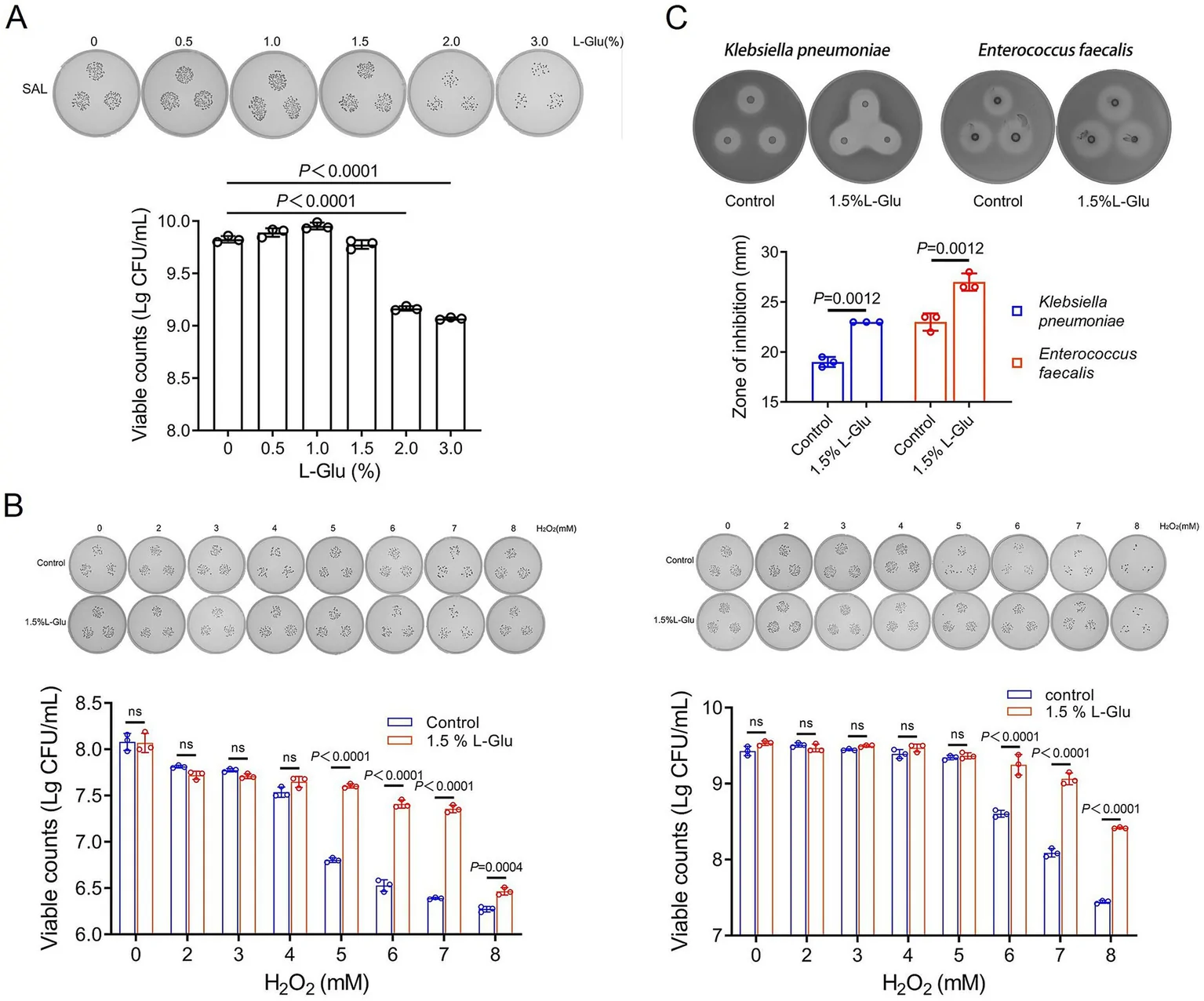
Protective effect of L-glutamate (L-Glu) supplementation on *L. plantarum* SAL under oxidative stress. **(A)** Growth of *L. plantarum* SAL in MRS broth supplemented with increasing concentrations of L-Glu (0–3%, w/v) after 12 h of incubation. **(B)** Viable counts of *L. plantarum* SAL cultured for 4 h (left) or 12 h (right) in the presence or absence of 1.5% L-Glu, followed by 0.5 h exposure to increasing concentrations of H_2_O_2_ (0–8 mM). **(C)** Antibacterial activity of *L. plantarum* SAL cultured with or without 1.5% L-Glu against *Klebsiella pneumoniae* and *Enterococcus faecalis*, measured as diameter of inhibition zones. Data are mean ± SD (*n* = 3). For **A**: One-way ANOVA with Tukey’s test; for **B**: Two-way ANOVA with Sidak’s test; for **C**: Unpaired *t*-test with Welch’s correction. Significance as in Figure 1.

After 4 h of culture (when *L. plantarum* SAL entered the logarithmic phase, strong proliferation, but the biological activity was not fully developed), no significant difference in viable counts was observed between the L-Glu-supplemented and nonsupplemented groups at H_2_O_2_ concentrations ≤ 3 mM. As the H_2_O_2_ concentration increased further, the counts in the control group (No L-Glu) decreased sharply, indicating a protective effect of L-Glu. Significant differences emerged at 5 mM H_2_O_2_. At 8 mM H_2_O_2_, the concentration in the 1.5% L-Glu group decreased to ~10^6^ CFU/mL but was still markedly greater than that in the control group (Figure 5B, left). After 12 h of culture (late logarithmic phase, proliferation slowing, and biological activity stabilizing), no significant difference was observed at H_2_O_2_ concentrations ≤ 5 mM. At 6 mM H_2_O_2_, the concentration in the control group decreased from ~10^9^ to ~10^8^ CFU/mL, whereas that in the 1.5% L-Glu group remained at ~10^9^ CFU/mL. Only at 8 mM H_2_O_2_ did the concentration in the 1.5% L-Glu group decrease to ~10^8^ CFU/mL, whereas that in the control group had already decreased to ~10^7^ CFU/mL (Figure 5B, right).

Compared with the control group cultured without L-Glu, *L. plantarum* SAL cultured in medium supplemented with 1.5% L-Glu exhibited increased antibacterial activity against *Klebsiella pneumoniae* and *Enterococcus faecalis* (Figure 5C). Therefore, supplementing the medium with 1.5% L-Glu not only maintained but also potentially improved the antibacterial activity of *L. plantarum* SAL.

### 3.6 Repair of intestinal barrier function in oxidatively damaged mice by *Lactiplantibacillus plantarum* SAL + L-Glu synbiotic

The experimental workflow is depicted in Figure 6A. After two weeks of intraperitoneal D-gal administration, the plasma levels of malondialdehyde (MDA) and total superoxide dismutase (T-SOD) activity significantly increased in the model group, confirming the successful establishment of the oxidative damage mouse model (Figure 6B). The effects of the synbiotic on body weight and colon length in oxidatively damaged mice were evaluated. Body weight and colon length were markedly lower in the D-gal model group than in the normal group. Compared with those in the D-gal model group, body weight and colon length were markedly greater in the SAL, L-Glu, and Synbiotic groups (Figure 6C). Hematoxylin and eosin (H&E) staining of the colon tissue revealed severe mucosal damage in the D-gal model group, characterized by broken villi, disorganized crypts, reduced goblet cell numbers, and significant inflammatory cell infiltration. Intervention with *L. plantarum* SAL markedly improved mucosal structure, increased the number of goblet cells, and reduced the severity of inflammation. L-Glu intervention also alleviated oxidative stress-induced structural damage, although the reparative effect was relatively weak. The Synbiotic group exhibited the most significant restoration: the villi were regularly arranged, the number of goblet cells was high, the mucosal tissue density approached normal, and the number of inflammatory cells was substantially reduced. These findings suggest synergistic effects between *L. plantarum* SAL and L-Glu in repairing intestinal barrier damage caused by oxidative stress (Figure 6D).

**Figure 6:**
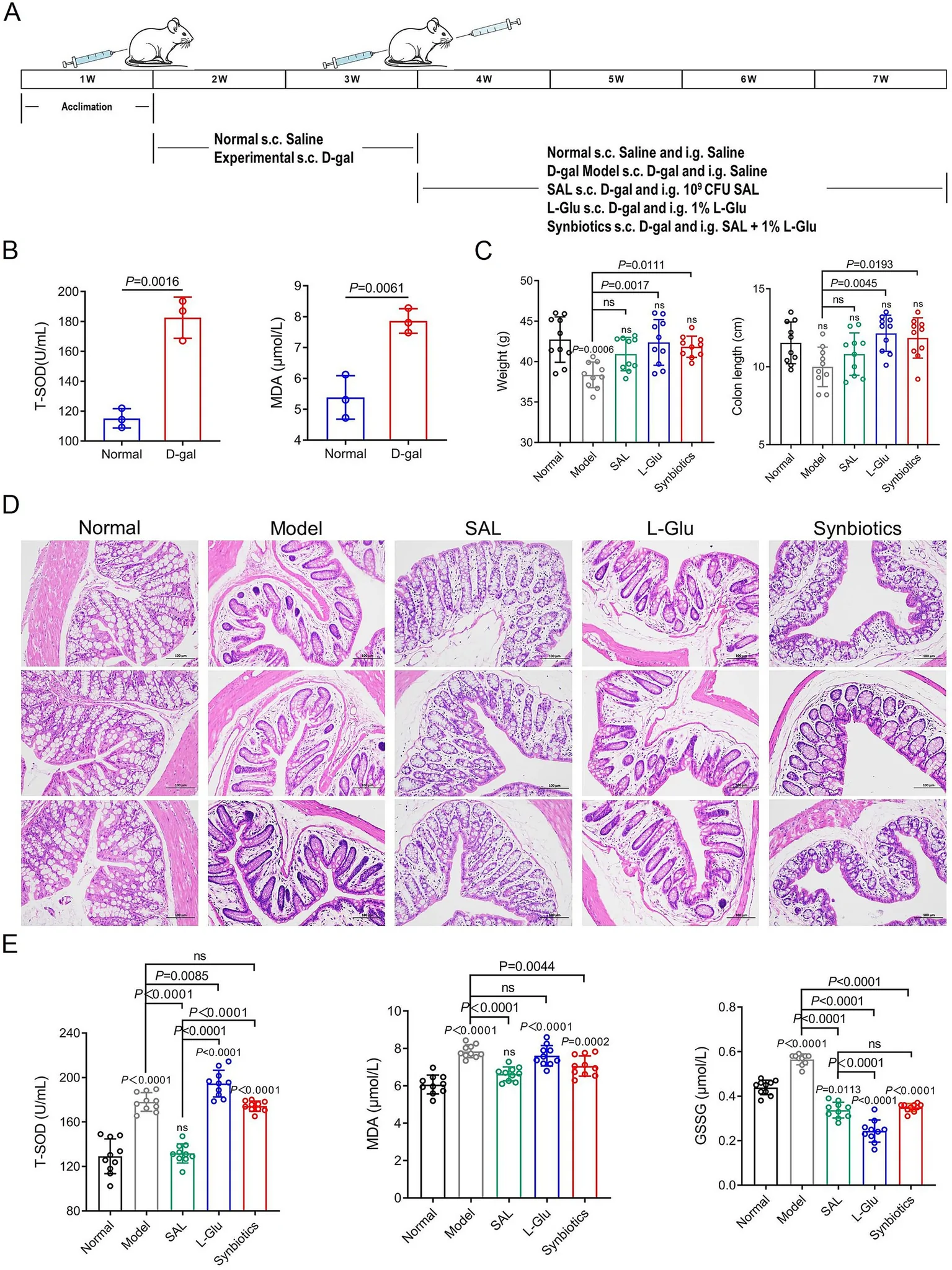
Restoration of intestinal barrier function by the *L. plantarum* SAL + L-Glu synbiotic in a D-galactose-induced oxidative damage mouse model. **(A)** Experimental workflow. **(B)** Plasma levels of malondialdehyde (MDA) and total superoxide dismutase (T-SOD) activity after two weeks of D-gal injection, confirming successful model establishment. **(C)** Body weight and colon length of mice after 4 weeks of treatment. **(D)** Representative H&E-stained colon tissue sections from each group. Scale bars (white lines, lower right corner of each image) represent 100 μm. **(E)** Plasma oxidative stress markers: MDA level, oxidized glutathione (GSSG) level, and T-SOD activity after 4 weeks of treatment. Data are mean ± SD (*n* = 10 for **C** and **E**; *n* = 3 for B). For **B**: Unpaired *t*-test; for **C** and **E**, one-way ANOVA with Tukey’s test. Significance as in Figure 1.

Compared with those in the normal group, the plasma levels of MDA and oxidized glutathione (GSSG), as well as T-SOD activity, were substantially elevated in the D-gal model group. Compared with those in the D-gal model group, the MDA, GSSG, and T-SOD levels in the SAL group were substantially lower. The L-Glu group showed a significant reduction only in GSSG levels. Compared with those in the D-gal group, the MDA, GSSG, and T-SOD levels in the synbiotic intervention group were substantially lower, and the T-SOD activity was markedly lower than in the D-gal group. Compared with those in the D-gal group, the MDA levels in the L-Glu group tended to decrease but did not significantly differ. Importantly, compared with the normal group, the synbiotic group exhibited no significant differences in the levels of these oxidative stress markers, indicating that redox homeostasis was restored (Figure 6E).

## 4 Discussion

The probiotic properties of *L. plantarum* SAL were systematically evaluated against the reference strain WCFS1. SAL exhibited significantly superior salt tolerance, maintaining markedly higher viability at 6% NaCl. This resilience likely reflects the presence of transporter clusters that accumulate compatible solutes under hyperosmotic stress, as well as genes encoding membrane proteins involved in Na^+^/K^+^ ion transport regulation (15). Both strains demonstrated comparable bile salt tolerance, an attribute attributed to bile salt hydrolase activity that hydrolyzes conjugated bile salts and mitigates their toxicity (16). Notably, SAL displayed significantly greater acid tolerance than WCFS1 in simulated gastric fluid at pH 1.5, consistent with the presence of an active GAD system (17). Both strains exhibited broad-spectrum antibacterial activity against Gram-negative and Gram-positive pathogens, likely mediated by organic acids, bacteriocins, and siderophores (18, 19).

In the CT26 colon cancer xenograft model, SAL administration significantly suppressed tumor growth (inhibition rates of 21.3% by weight and 32.5% by volume) and attenuated splenomegaly, effects comparable to those of WCFS1. Both strains elevated serum levels of TNF-α, TNF-β, and IFN-γ, reflecting enhanced Th1-type immune responses and immune surveillance (20, 21). Western blot analysis revealed that both strains promoted tumor cell apoptosis by upregulating the proapoptotic regulators Bax and Caspase-3 while downregulating the antiapoptotic protein Bcl-2 (11). These findings confirm that SAL possesses potent antitumor and immunomodulatory activities, desirable traits for a probiotic candidate.

The finding that *gad*B expression enhances oxidative stress tolerance in *E. coli* represents a conceptual advance. While GAD has been extensively studied for its role in acid resistance (9), its contribution to oxidative stress defense has received limited attention (13). Transcriptomic profiling of H_2_O_2_-challenged SAL identified 313 differentially expressed genes, with *gad*B and *pur*A emerging as key regulatory hubs. Heterologous expression of recombinant *Gad*B (specific activity 14.1 U/mg) conferred marked oxidative stress resistance to *E. coli*, demonstrating that *Gad*B-mediated GABA biosynthesis provides cross-protection against oxidant challenge. This dual-protection model extends the known function of the GAD system beyond acid resistance (22): GAD both consumes protons to maintain pH homeostasis and reroutes glutamate metabolism toward GABA, which enters the TCA cycle via the GABA shunt to sustain energy production and redox balance (23, 24). Distinct from previous studies that characterized GAD primarily as an acid-resistance system in other lactic acid bacteria, the present work demonstrates that *gad*B upregulation occurs specifically under H_2_O_2_ stress and confers cross-protection against oxidation, suggesting a broader physiological role for this metabolic pathway in probiotic stress adaptation.

Comparisons with previous GAD-focused studies underscore the distinct contribution of the findings. Cataldo et al. (12) and Yunes et al. (14) characterized the genetic and regulatory basis of GABA production in gut commensals, but their work was confined to acid resistance and did not address oxidative stress or provide heterologous functional validation. The present data demonstrate that *gad*B upregulation occurs specifically under H_2_O_2_ stress and that *Gad*B alone is sufficient to confer oxidant tolerance, a cross-protective function not previously reported in *Lactiplantibacillus*. Moreover, unlike exogenous GABA supplementation (22), the substrate-targeted synbiotic (SAL + L-Glu) enables continuous *in situ* GABA production at the mucosal interface, coupling bacterial metabolism with host substrate availability for sustained local delivery. Collectively, these advances extend the canonical GAD acid-resistance paradigm into a broader oxidative stress defense framework with direct translational relevance.

*Gad*B-mediated protection involves several interconnected processes (Figure 4G). Under H_2_O_2_ exposure, increased intracellular H^+^ concentration activates *gad*B transcription and promotes translocation of GAD to the cell membrane (12). Membrane-associated GAD catalyzes the decarboxylation of L-Glu to GABA, consuming one intracellular proton per GABA molecule generated (17). This continuous proton consumption helps maintain cytoplasmic pH homeostasis. Concurrently, the GadC antiporter exports accumulated GABA from the cytoplasm, reducing metabolic burden (9). The exported GABA can undergo transamination catalyzed by GABA transaminase, producing succinic semialdehyde (SSA) and transferring its amino group to α-ketoglutarate to form glutamate. SSA is subsequently dehydrogenated by succinate semialdehyde dehydrogenase (SSADH) to produce succinate, which enters the TCA cycle and supports ATP production. Thus, by enhancing GABA metabolic flux, GAD enables bacteria to maintain energy supply and stress resistance under oxidative stress conditions (25).

Although the present study did not directly measure Nrf2 activation or tight junction proteins in intestinal epithelia, the observed *in vivo* phenotype (restored T-SOD activity and reduced MDA/GSSG levels) closely parallels the Keap1/Nrf2/HO-1 axis activation documented for Lactiplantibacillus strains (13, 26–28). γ-Aminobutyric acid (GABA), the direct enzymatic product of *Gad*B, has been independently validated as a Nrf2 agonist that induces phase II antioxidant enzymes in mammalian cells (29, 30). Based on these published findings and the consistency of the redox data with the Nrf2-driven antioxidant response, a mechanistic model is proposed in which *Gad*B-derived GABA, amplified by exogenous L-Glu, triggers Nrf2 nuclear translocation and ARE-dependent transcription of SOD, GPx, and catalase, thereby accounting for the restored redox balance and mucosal repair observed in synbiotic-treated mice. Direct verification of Nrf2 activation in this specific experimental context remains an important objective for future research. Beyond its canonical acid-tolerance function, the GAD/GABA system thus appears to function as a metabolic signaling axis that couples bacterial metabolism to host redox homeostasis, revealing a previously underappreciated dimension of probiotic–host crosstalk.

L-Glu supplementation (1.5%) significantly increased the tolerance of SAL to H_2_O_2_-induced oxidative stress, maintaining viable counts at ~10^8^ CFU/mL under 8 mM H_2_O_2_ versus ~10^7^ CFU/mL without supplementation. This protective effect is likely dependent on GAD system activation, as L-Glu serves as the direct substrate for *Gad*B-catalyzed decarboxylation. In the D-gal-induced oxidative damage model, the SAL + L-Glu synbiotic restored colon barrier integrity, increased goblet cell numbers, reduced inflammatory infiltration, and decreased plasma MDA and GSSG levels to values comparable to those of normal controls. The synergistic mechanism likely involves multilayered metabolic complementarity: (i) SAL decarboxylates L-Glu to GABA via *Gad*B, activating antioxidant responses and maintaining pH homeostasis (31); (ii) SAL exerts anti-inflammatory effects; and (iii) L-Glu serves as a glutathione precursor, providing substrate support for the antioxidant system (32). The observation that L-Glu alone failed to reduce MDA levels suggests a hormetic dose–response profile for this amino acid. At low-to-moderate concentrations, L-Glu serves as a GSH precursor and *Gad*B substrate; however, at high concentrations, its metal-chelating properties may promote Fe^2+^-driven Fenton chemistry (33). This biphasic effect underscores the importance of dose optimization in synbiotic formulations and argues against simple “more is better” supplementation strategies.

Several study limitations should be acknowledged. First, although the GABA-Nrf2 axis is proposed as a literature-supported mechanistic inference based on the observed *in vivo* phenotype and previous reports, direct molecular verification of Nrf2 activation within this specific experimental setting remains an avenue for future investigation. Second, the optimal L-Glu dosage was selected on the basis of preliminary *in vitro* experiments and food additive safety standards, but systematic dose optimization *in vivo* has not been performed; the potential pro-oxidant risk of high-dose L-Glu requires careful evaluation in future translational studies. Third, the synbiotic efficacy was validated in the D-gal model; whether similar protective effects occur in other oxidative stress models, such as DSS-induced colitis, radiation injury, or metabolic syndrome, remains to be determined. Fourth, while the present study demonstrates the protective effects of the synbiotic at the bacterial and organismal levels, the intermediate cellular responses, including whether GABA directly protects intestinal epithelial cells from oxidative injury or whether the effect is mediated through microbial metabolites, were not examined *in vitro*. This omission was intentional: the primary objective of this study was to establish the bacterial stress-response mechanism (*gad*B identification and validation in *E. coli*) and its *in vivo* translational proof-of-concept in a well-established oxidative damage model. A full-scale epithelial cell mechanistic study, though valuable, was not undertaken here, because such reductionist systems cannot recapitulate the multi-factor interactions (bacterial colonization, mucus penetration, and host-microbe-substrate crosstalk) that are central to synbiotic function *in vivo*. Accordingly, priority was given to whole-organism efficacy in a well-established oxidative damage model as the translational proof-of-concept. Nevertheless, such investigations are planned as a logical next step, with future studies designed to include assessments of tight junction proteins (occludin, ZO-1), Nrf2 nuclear translocation, and intracellular ROS levels in epithelial cells treated with GABA and L-Glu, which will help delineate the direct epithelial protective mechanisms underlying the symbiotic’s effects. Despite these limitations, the present findings establish *gad*B as a key mediator of oxidative stress cross-protection in *L. plantarum* SAL and demonstrate the feasibility of substrate-targeted synbiotic strategies for intestinal barrier protection.

## 5 Conclusion

This study demonstrated that *L. plantarum* SAL possesses sophisticated stress adaptation mechanisms and engages in synergistic metabolic interactions with L-glutamate, substantially enhancing its ability to remodel the gut microenvironment and restore redox homeostasis. Transcriptomic analysis revealed *gad*B as a key gene critical for oxidative stress resistance. Subsequent validation using an *E. coli* prokaryotic expression system confirmed that the GAD system confers exceptional acid resistance by consuming H^+^ during GABA synthesis. Importantly, supplementation with L-Glu, the substrate for the *gad*B pathway, resulted in a synergistic synbiotic combination that significantly ameliorated D-galactose-induced oxidative damage in mice. These findings underscore the significant potential of *L. plantarum* SAL as an adjuvant therapeutic agent for reconstructing gut microecology. While the synbiotic showed robust efficacy, the precise molecular crosstalk between *Gad*B-mediated GABA production and host Nrf2 pathway activation requires further mechanistic dissection. Additionally, the potential pro-oxidant effects of high-dose L-glutamate warrant careful dose optimization in future translational studies.

## Data Availability

The raw RNA-sequencing data reported in this paper have been deposited in the Genome Sequence Archive in the National Genomics Data Center, China National Center for Bioinformation/Beijing Institute of Genomics, Chinese Academy of Sciences (GSA: CRA027909), and are publicly accessible at: https://ngdc.cncb.ac.cn/gsa. Other data that support the findings of this study are available from the corresponding author upon reasonable request.

## Footnotes

1
http://Metascape.org

2
http://string-db.org

## Glossary

Bax: Bcl-2-associated X protein
Bcl-2: B-cell lymphoma 2 protein
BSH: Bile salt hydrolase
CFU: Colony-forming unit
D-gal: D-galactose
DEGs: Differentially expressed genes
ELISA: Enzyme-linked immunosorbent assay
GABA: γ-aminobutyric acid
GAD: Glutamate decarboxylase
GAPDH: Glyceraldehyde-3-phosphate dehydrogenase
GO: Gene Ontology
GPx: Glutathione peroxidase
GSH: Glutathione
GSR: Glutathione reductase
GSSG: Oxidized glutathione
H&E: Hematoxylin and eosin
HPLC: High-performance liquid chromatography
IFN-γ: Interferon-γ
IPTG: Isopropyl β-D-1-thiogalactopyranoside
KEGG: Kyoto Encyclopedia of Genes and Genomes
L-Glu: L-glutamate
LB: Luria-Bertani
MDA: Malondialdehyde
MRS: De Man, Rogosa and Sharpe
Nrf2: Nuclear factor erythroid 2-related factor 2
PLP: Pyridoxal 5′-phosphate
PPI: Protein–protein interaction
qRT-PCR: Quantitative real-time PCR
ROS: Reactive oxygen species
SCFAs: Short-chain fatty acids
SDS-PAGE: Sodium dodecyl sulfate-polyacrylamide gel electrophoresis
SOD: Superoxide dismutase
SPF: Specific pathogen-free
SSA: Succinic semialdehyde
TNF-α: Tumor necrosis factor-α
TNF-β: Tumor necrosis factor-β
T-SOD: Total superoxide dismutase
